# Association between changes in corrected anion gap and mortality among critically ill patients during ICU stay: a multicenter observational study

**DOI:** 10.3389/fphys.2025.1469985

**Published:** 2025-06-25

**Authors:** Yanli Hou, Ruohan Li, Jiamei Li, Jingjing Zhang, Jiajia Ren, Ya Gao, Xuting Jin, Yanni Luo, Xiaochuang Wang, Gang Wang

**Affiliations:** ^1^ Department of Critical Care Medicine, The Second Affiliated Hospital of Xi’an Jiaotong University, Xi’an, Shaanxi, China; ^2^ Key Laboratory of Surgical Critical Care and Life Support (Xi’an Jiaotong University), Ministry of Education, Xi’an, Shaanxi, China

**Keywords:** changes in corrected anion gap, mortality, intensive care unit, eICU Collaborative Research Database, retrospective cohort study

## Abstract

**Background:**

The research on the impact of dynamic corrected anion gap (cAG) on prognosis is scarce.

**Objective:**

This study aimed to investigate the relationship between changes in cAG (ΔcAG) during intensive care unit (ICU) hospitalization and mortality.

**Methods:**

In this multicenter, retrospective cohort study, patients with both initial and final records of serum sodium, potassium, chloride, bicarbonate, and albumin were recruited from the eICU Collaborative Research Database. Two cohorts were included in the study: cohort A (final cAG > initial cAG) and cohort B (final cAG < initial cAG). Multivariable logistic regression was utilized to assess the association between mortality and ΔcAG in each cohort. ΔcAG was calculated as shown as follows: $\Delta cAG=\frac{\left|final\;cAG\;‐\;initial\;cAG\right|}{initial\;cAG}\times 100\%$ .

**Results:**

Among the 11,216 enrolled patients, 4,147 (37%) individuals were classified into cohort A, while 7,069 (63%) patients were assigned to cohort B. In cohort A, for every 10% increase in ΔcAG, ICU and hospital mortalities increased by 46.1% (odds ratio: 1.461, 95% confidence interval [1.378, 1.548]) and 55.5% (1.555 [1.467, 1.648]), respectively. Interaction and subgroup analyses demonstrated consistent results among patients with different Acute Physiology and Chronic Health Evaluation Ⅳ (APACHE Ⅳ) scores (≤58 vs. >58), time interval (≤97 h vs. >97 h) and initial cAG (≤16 mEq/L vs. >16 mEq/L). Meanwhile, in cohort B, ICU and hospital mortalities decreased by 31.4% (0.686 [0.619, 0.759]) and 29.4% (0.706 [0.651, 0.764]), respectively, with each 10% increase in ΔcAG, especially among patients with higher APACHE IV scores (>62) and initial cAG (>16 mEq/L). When analyzed categorically, the ΔcAG still exhibited a significant risk gradient across quartiles.

**Conclusion:**

Further elevated cAG after ICU admission demonstrates a robust association with an increased mortality risk in critically ill patients. ICU patients with higher APACHE Ⅳ scores or initial cAG may benefit from measures aimed at reducing cAG.

## 1 Introduction

Critically ill patients commonly experience a variety of acid-base disorders (Achanti and Szerlip, 2023; Cerdá et al., 2012). Among these, acute metabolic acidosis is recognized as one of the most severe forms (Yagi and Fujii, 2021). This condition exerts multisystem effects, including diminished cardiac contractility and reduced cardiac output, induced arterial vasodilation, and neurological manifestations such as mental confusion and lethargy. These pathophysiological alterations promote systemic inflammation, compromise immune response, and trigger cellular death (Kraut and Madias, 2010). The anion gap (AG) is routinely employed to estimate the difference between measured and unmeasured major extracellular fluid cations and anions, helping to identify the underlying cause of metabolic acidosis (Kraut and Madias, 2010). Possible causes include lactic acidosis, ketoacidosis, and uremic acidosis; ingestion of salicylate, methanol, ethylene glycol, or propylene glycol; and many inborn errors of metabolism (Goodkin et al., 1984). However, hypoalbuminemia, which is commonly observed in critically ill patients, can lower the AG and mask an acidosis. Albumin-corrected anion gap (cAG) is a more suitable screening tool for the diagnosis of metabolic acidosis in intensive care unit (ICU) (Hatherill et al., 2002).

Previous studies have indicated that elevated levels of cAG upon ICU admission serve as prognostic indicators for mortality in patients with sepsis (Hu et al., 2021), chronic obstructive pulmonary disease (Giri et al., 2025), acute pesticide poisoning (Lee et al., 2019), and severe cardiac disease (Zhao et al., 2023). However, conflicting evidence suggested that cAG may not reliably predict hospital mortality in critically ill patients, as indicated by small areas under the receiver operating characteristic curve (AUROC) values (Rocktaeschel et al., 2003). Therefore, previous studies have primarily focused on examining the relationship between baseline cAG levels and prognosis, and findings have been somewhat controversial. Given the dynamic and rapidly evolving clinical status of ICU patients, it is necessary to further explore the correlation between prognosis and cAG by utilizing a relative dynamic index in this patient population.

A published retrospective study involving 18,985 critically ill patients demonstrated that an elevation in AG between prehospital admission and critical care initiation predicted the risk of all-cause mortality in this population (Lipnick et al., 2013). Similarly, another retrospective cohort study found that the change in cAG (ΔcAG) during the first 3 days after ICU admission was a prognostic indicator for hospital mortality and 90-day overall survival outcomes (Xie et al., 2022). However, these studies defined ΔcAG as changes occurring over relatively short period during the ICU stay. More investigations are warranted to explore the relationship between ΔcAG occurring over an extended duration and its impact on patient prognosis. Therefore, this study aimed to evaluate the association between ΔcAG during ICU hospitalization and mortality among critically ill patients, utilizing data from the eICU Collaborative Research Database (eICU-CRD, version 2.0).

## 2 Materials and methods

### 2.1 Data description

This multicenter observational cohort study employed the publicly available eICU-CRD version 2.0, a de-identified ICU database comprising 139,367 unique patients admitted to 335 units across 208 hospitals in the United States between 2014 and 2015 (Pollard et al., 2018). The database is maintained by the Laboratory for Computational Physiology at the Massachusetts Institute of Technology (MIT; Cambridge, MA, United States) and can be accessed at https://physionet.org/content/eicu-crd/. The eICU database was released under the Health Insurance Portability and Accountability Act safe harbor provision, and all protected health information was de-identified. Hence individual patient consent was not required. Furthermore, all authors of the manuscript underwent the required training and obtained permission to access the database.

### 2.2 Data extraction

We extracted patient data from the eICU-CRD using SAS version 9.4 (SAS Institute, Cary, NC). The extracted data encompassed demographic records, clinical comorbidities at ICU admission, administered treatments, severity-of-illness scores as assessed by the Acute Physiology and Chronic Health Evaluation Ⅳ (APACHE Ⅳ) score, ICU length of stay, discharge status from the ICU and hospital, and comprehensive records of biochemical indicators at both ICU admission and discharge. Demographic information included age, sex, and ethnicity. Clinical comorbidities were identified using the International Classification of Diseases-9 (ICD-9) codes and comprised metabolic derangements (e.g., acidosis, alkalosis and mixed acid base disorder), heart failure, respiratory failure, renal failure, disseminated intravascular coagulation (DIC), diabetes, shock, tumor, trauma, sepsis, and hepatic disease. Treatments of interest included catecholamine and diuretic therapy, fluid resuscitation, mechanical ventilation, and dialysis during the ICU stay. The dataset also included initial records of serum sodium (Na^+^), potassium (K^+^), chloride (Cl^−^), bicarbonate (HCO_3_^−^), blood pH, serum creatinine, and serum lactate levels after ICU admission. Additionally, the final records of Na^+^, K^+^, Cl^−^, and HCO_3_^−^ levels before ICU discharge were included. Serum albumin concentrations were measured within 24 h of the recording of Na^+^, K^+^, Cl^−^, and HCO_3_^−^ levels.

### 2.3 ΔcAG and outcome

We calculated AG using the formula: *AG* = ([*Na* ^*+*^] mEq/L + [*K* ^*+*^] mEq/L) - ([*Cl* ^*−*^] mEq/L + [*HCO*_*3*_^*−*^] mEq/L) (Seifter, 2014). To account for the influence of abnormal albumin concentration, the corrected AG was derived as: *cAG* = *AG* + 2.5 × [4.4 - *albumin* (g/dL)] (Figge et al., 1998). The exposure of interest was ΔcAG, calculated as: $\Delta cAG=\frac{\left|final\;cAG\;‐\;initial\;cAG\right|}{initial\;cAG}\times 100\%$ . Moreover, the absolute change in cAG (cAG_Diff_) was calculated as: ${cAG}_{Diff\;}=|final\;cAG\;‐\;initial\;cAG|$ . The initial cAG was defined the value calculated from the first records of Na^+^, K^+^, Cl^−^, HCO_3_^−^, and albumin after ICU admission. The final cAG was calculated using the last available measurement before ICU discharge, with a minimum time interval of 24 h from the initial record. The primary outcome was ICU mortality, defined as death occurring before ICU discharge for any cause. The secondary outcome was hospital mortality, defined as death from any cause occurring before hospital discharge.

### 2.4 Patient selection

In this study, patient information was exclusively sourced from the eICU-CRD. The inclusion criteria were as follows: (1) single hospital admission; (2) first ICU admission; (3) an ICU length of stay >24 h; (4) age ≥15 years old; (5) at least two records of Na^+^, K^+^, Cl^−^, and HCO_3_^−^. Patients meeting any of the following exclusion criteria were excluded: (1) those with disparate timings for initial and/or final records of Na^+^, K^+^, Cl^−^, or HCO_3_^−^ levels; (2) those lacking initial and/or final records of albumin; (3) those with a time interval less than 24 h between the initial and final recording of Na^+^, K^+^, Cl^−^, HCO_3_^−^, or albumin; (4) those with missing or unqualified covariates for multivariable adjustment; and (5) those without recorded ICU discharge status. Based on the comparison between final cAG and initial cAG, the study population was stratified into two cohorts: cohort A (final cAG > initial cAG) and cohort B (final cAG < initial cAG).

### 2.5 Statistical analysis

Continuous variables were expressed as medians with interquartile ranges (IQRs) and compared using the Kruskal‒Wallis H test. Categorical variables were presented as frequencies (percentages) and analyzed through the χ^2^ test or Fisher’s exact test. A Chord diagram was generated to visually illustrate the correlation between initial cAG and final cAG for each patient. Logistic regression models were employed to evaluate the associations between ΔcAG or cAG_Diff_ and ICU and hospital mortality risks. Multivariable adjustments included demographic information, time interval between initial and final cAG measurements, treatments, biochemical indicators, and clinical comorbidities. For missing data on lactate and pH values, multivariate imputation by chained equations (MICE) was performed utilizing the MICE package in the R project (Zhang, 2016) to ensure a more complete dataset for analysis. To explain multicollinearity, variance inflation factors (VIFs) were calculated to assess variables in the multivariable model. A *VIF* < 10 indicates that multicollinearity may not affect the estimation (Kutner et al., 2004). Restricted cubic splines (RCS) were used to graphically portray the relationship between ΔcAG and mortality. A two-sided *P* < 0.05 was considered statistically significant.

Interaction and subgroup analyses were conducted *post hoc* to explore the potential consistency of the relationship between ΔcAG and mortality across different clinical contexts. Stratification was based on median values of time interval (between the initial and final cAG) or APACHE Ⅳ score, as well as the initial cAG value of 16 mEq/L (Goldstein and Halperin, 2010; Oh, 2011). To mitigate false discovery risks, we applied Bonferroni correction (*adjusted α* = 0.05/2) to all subgroup comparisons performed. All statistical analyses were performed using SPSS version 22.0 (SPSS, Chicago, IL, United States).

## 3 Results

### 3.1 Individual selection and clinical characteristics

**FIGURE 1:**
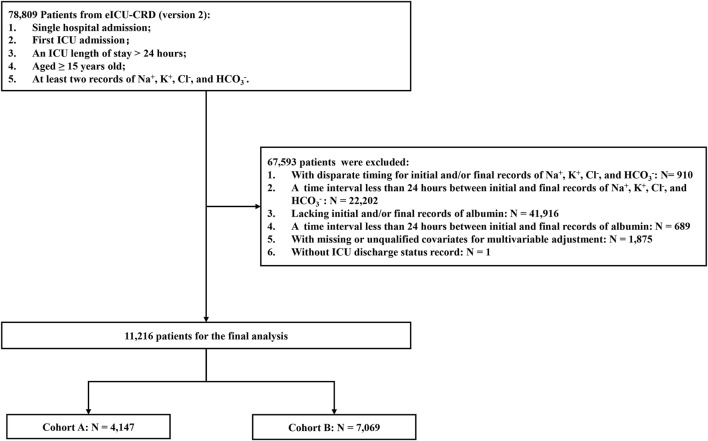
Flow diagram of participant selection. Cohort selection and criteria for exclusion: a total of 11,216 patients were included in the analysis. Among them, 4,147 patients experienced an elevation, while 7,069 patients experienced a reduction in their final cAG level compared with that at initial measurement. eICU-CRD, eICU Collaborative Research Database; ICU, intensive care unit; cAG, corrected anion gap.

A total of 11,216 patients entered the final cohort. Among them, 4,147 patients exhibited an elevated final cAG level compared to the initial measurement (cohort A), while 7,069 patients showed a decrease in final cAG level (cohort B) (Figure 1). The Chord diagram depicts the relationship between the final cAG and initial cAG for each patient (Supplementary Figure 1). Of the cohort A, 2,350 participants (56.7%) were male, 3,204 (77.3%) were Caucasian, and 1,942 (46.8%) were aged >65 years. ICU and hospital mortality rates were 14.6% and 20.9%, respectively. The time interval between the final and initial cAG was 97 (50–172) hours, with an initial cAG of 15.75 (13.55–18.45) mEq/L and an APACHE Ⅳ score of 58 (42–78). We stratified the study cohort based on quartiles of ΔcAG ≤5.34%, 5.34% < ΔcAG ≤12.32%, 12.32% < ΔcAG ≤23.70%, and ΔcAG >23.70% (Table 1). Among 7,069 patients in cohort B, 54.8% were male, with a predominant Caucasian ethnicity (77.1%) and 45.2% aged >65 years. ICU and hospital mortality rates were 7.2% and 12.2%, respectively. The time interval between the final and initial cAG was 101 (55–194) hours, with an initial cAG of 19.10 (16.65–22.15) mEq/L and an APACHE Ⅳ score of 62 (46–81). The cohort was stratified into ΔcAG quartiles: ΔcAG ≤7.48%, 7.48%, < ΔcAG ≤15.05%, 15.05% < ΔcAG ≤24.70%, and ΔcAG >24.70% (Supplementary Table 1).

**TABLE 1:** Baseline characteristics of Cohort A.

Characteristics	Entire population (N = 4,147)	ΔcAG (%)				
		Q1: ≤5.34% (N = 1,037)	Q2: 5.34%–12.32% (N = 1,037)	Q4: 12.32%–23.70% (N = 1,037)	Q4: >23.70% (N = 1,036)	P value
Age n (%)						0.863
≤65 years	2,205 (53.2)	543 (52.4)	552 (53.2)	562 (54.2)	548 (52.9)	
>65 years	1,942 (46.8)	494 (47.6)	485 (46.8)	475 (45.8)	488 (47.1)	
Sex n (%)						0.403
Male	2,350 (56.7)	565 (54.5)	592 (57.1)	592 (57.1)	601 (58.0)	
Female	1,797 (43.3)	472 (45.5)	445 (42.9)	445 (42.9)	435 (42.0)	
Ethnicity n (%)						0.175
Caucasian	3,204 (77.3)	819 (79.0)	793 (76.5)	781 (75.3)	811 (78.3)	
Others/Unknown	943 (22.7)	218 (21.0)	244 (23.5)	256 (24.7)	225 (21.7)	
APACHE Ⅳ score	58 (42–78)	57 (40–74)	56 (40–75)	56 (41–76)	62 (46–86)	<0.001
Initial cAG (mEq/L)	15.75 (13.55–18.45)	16.80 (14.65–19.15)	16.00 (14.15–18.40)	15.15 (13.38–17.70)	14.80 (12.36–12.09)	<0.001
Time Interval (hours)	97 (50–172)	90 (49–161)	96 (50–177)	94 (50–152)	109 (59–214)	<0.001
pH value	7.38 (7.33–7.43)	7.38 (7.33–7.43)	7.39 (7.33–7.43)	7.38 (7.33–7.43)	7.37 (7.32–7.43)	0.084
Lactate (mmol/L)	1.50 (1.00–2.20)	1.57 (1.00–2.20)	1.40 (1.00–2.10)	1.40 (1.00–2.20)	1.50 (1.00–2.60)	<0.001
Creatinine (mg/dL)	1.00 (0.73–1.64)	1.00 (0.73–1.64)	0.96 (0.70–1.58)	0.96 (0.73–1.50)	1.08 (0.77–2.82)	0.001
Treatments n (%)						
Catecholamine	909 (21.9)	187 (18.0)	202 (19.5)	223 (21.5)	297 (28.7)	<0.001
Dialysis	332 (8.0)	59 (5.7)	62 (6.0)	70 (6.8)	141 (13.6)	<0.001
Mechanical Ventilation	1929 (46.5)	432 (41.7)	482 (46.5)	462 (44.6)	553 (53.4)	<0.001
fluid resuscitation	225 (5.4)	68 (6.6)	47 (4.5)	56 (5.4)	54 (5.2)	0.231
Diuretic	915 (22.1)	206 (19.9)	217 (20.9)	223 (21.5)	269 (26.0)	0.005
Diagnoses n (%)						
Metabolic derangements	162 (3.9)	38 (3.7)	43 (4.1)	32 (3.1)	49 (4.7)	0.256
Heart Failure	328 (7.9)	79 (7.6)	92 (8.9)	75 (7.2)	82 (7.9)	0.554
Renal Failure	488 (11.8)	118 (11.4)	116 (11.2)	120 (11.6)	134 (12.9)	0.597
Respiratory Failure	1,010 (24.4)	225 (21.7)	230 (22.2)	257 (24.8)	298 (28.8)	0.001
DIC	13 (0.30)	1 (0.1)	3 (0.3)	5 (0.5)	4 (0.4)	0.417
Diabetes	399 (9.6)	94 (9.1)	114 (11.0)	100 (9.6)	91 (8.8)	0.327
Shock	931 (22.4)	218 (21.0)	229 (22.1)	233 (22.5)	251 (24.2)	0.365
Tumor	320 (7.7)	79 (7.6)	75 (7.2)	78 (7.5)	88 (8.5)	0.731
Trauma	280 (6.8)	57 (5.5)	70 (6.8)	69 (6.7)	84 (8.1)	0.131
Sepsis	791 (19.1)	205 (19.8)	187 (18.0)	204 (19.7)	195 (18.8)	0.721
Hepatic Disease	364 (8.8)	78 (7.5)	82 (7.9)	93 (9.0)	111 (10.7)	0.048
ICU Mortality	606 (14.6)	90 (8.7)	97 (9.4)	126 (12.2)	293 (28.3)	<0.001
Hospital Mortality	867 (20.9)	136 (13.1)	152 (14.7)	194 (18.7)	385 (37.2)	<0.001

cAG, corrected anion gap; ΔcAG, changes in corrected anion gap, APACHE Ⅳ, Acute Physiology and Chronic Health Evaluation Ⅳ; DIC, disseminated intravascular coagulation; ICU, intensive care unit. Time Interval, the time interval between the final and the initial cAG measurement; $\text{AG}=\left(\left[Na^{+}\right]\text{mEq}/L+\left[K^{+}\right]\text{mEq}/L\right)-\left(\left[Cl^{-}\right]\text{mEq}/L+\left[{\text{HCO}}_{3}^{-}\right]\text{mEq}/L\right);\text{ cAG}=\text{AG}+2.5\times \left[4.4-\text{albumin }\left(g/\text{dL}\right)\right]$ ; $\Delta cAG=\frac{\left|final\;cAG\;‐\;initial\;cAG\right|}{initial\;cAG}\times 100\%$ .

### 3.2 Association between ΔcAG and mortality

We incorporated ΔcAG/10 as a continuous variable in the multivariable analysis, adjusting for all potential confounders. The results revealed that in cohort A, for every 10% increase in ΔcAG, critically ill patients exhibited a 46.1% increase in ICU mortality (odds ratio (OR), 1.461; 95% confidence interval (CI), [1.378, 1.548]) and a 55.5% increase in hospital mortality (1.555 [1.467, 1.648]). Moreover, in cohort B, a 10% decrease in ΔcAG was associated with a significant reduction of 31.4% (0.686 [0.619, 0.759]) and 29.4% (0.706 [0.651, 0.764]) for ICU and hospital mortality, respectively.

**FIGURE 2:**
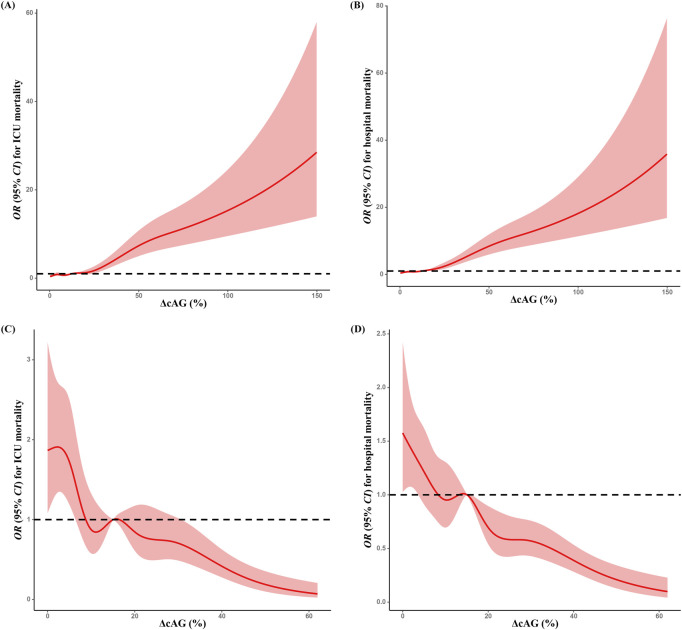
Restricted spline curves for the association between ΔcAG and mortality. Associations between ΔcAG^a^ and ICU **(A)** and hospital **(B)** mortalities in cohort A; associations between ΔcAG^a^ and ICU **(C)** and hospital **(D)** mortalities in cohort **(B)** All multivariable models were adjusted for demographic information (age [category], sex, ethnicity); APACHE Ⅳ score, biochemical indicators (pH, serum creatinine, lactate, and initial cAG); time interval (the hours between the final and the initial cAG measurement); treatments (catecholamine, dialysis, mechanical ventilation, fluid resuscitation, diuretic); clinical comorbidities (metabolic derangements, heart failure, renal failure, respiratory failure, DIC, Diabetes, shock, tumor, trauma, sepsis, hepatic disease). cAG, corrected anion gap; ΔcAG, changes in corrected anion gap, ICU, intensive care unit; APACHE Ⅳ, Acute Physiology and Chronic Health Evaluation Ⅳ; DIC, disseminated intravascular coagulation. ^a^: $\Delta cAG=\frac{\left|final\;cAG\;‐\;initial\;cAG\right|}{initial\;cAG}\times 100\%$

When considering ΔcAG as a categorical variable in multivariable logistic regression analysis, we observed higher risks of ICU (Q3 vs. Q1: 1.858 [1.302, 2.653]; Q4 vs. Q1: 6.483 [4.631, 9.076]) and hospital (Q2 vs. Q1: 1.403 [1.030, 1.911]; Q3 vs. Q1: 2.349 [1.733, 3.182]; Q4 vs. Q1: 7.798 [5.785, 10.513]) mortalities in the upper quartile among cohort A. In cohort B, the upper quartiles of ΔcAG had lower risks of ICU (Q2 vs. Q1: 0.556 [0.413, 0.749]; Q3 vs. Q1: 0.577 [0.432, 0.771]; Q4 vs. Q1: 0.344 [0.248, 0.478]) and hospital (Q2 vs. Q1: 0.777 [0.618, 0.976]; Q3 vs. Q1: 0.628 [0.499, 0.792]; Q4 vs. Q1: 0.388 [0.299, 0.503]) mortalities (Table 2). Additionally, RCS analysis revealed a positive association between the degree of ΔcAG and mortality in cohort A (Figures 2A,B), whereas a negative relationship was observed in cohort B (Figures 2C,D). The association was similar between the cAG_Diff_ and mortality (Supplementary Table 2).

**TABLE 2:** Multivariable analysis of the association between ΔcAG and mortality.

ΔcAG^a^ variable	ICU mortality		Hospital mortality	
	*OR* (95% *CI*)	*P* value	*OR* (95% *CI*)	*P* value
Cohort A				
Continuous variable^b^	1.461 (1.378, 1.548)	<0.001	1.555 (1.467, 1.648)	<0.001
Categorical variable				
Q1	1 (Ref)		1 (Ref)	
Q2	1.206 (0.835, 1.742)	0.318	1.403 (1.030, 1.911)	0.032
Q3	1.858 (1.302, 2.653)	0.001	2.349 (1.733, 3.182)	<0.001
Q4	6.483 (4.631, 9.076)	<0.001	7.798 (5.785, 10.513)	<0.001
Cohort B				
Continuous variable^b^	0.686 (0.619, 0.759)	<0.001	0.706 (0.651, 0.764)	<0.001
Categorical variable				
Q1	1 (Ref)		1 (Ref)	
Q2	0.556 (0.413, 0.749)	<0.001	0.777 (0.618, 0.976)	0.030
Q3	0.577 (0.432, 0.771)	<0.001	0.628 (0.499, 0.792)	<0.001
Q4	0.344 (0.248, 0.478)	<0.001	0.388 (0.299, 0.503)	<0.001

Multivariable model: Adjusted for demographic information (age [category], sex, ethnicity); APACHE Ⅳ score, biochemical indicators (pH, serum creatinine, lactate, and initial cAG); time interval (the hours between the final and the initial cAG measurement); treatments (catecholamine, dialysis, mechanical ventilation, fluid resuscitation, diuretic); clinical comorbidities (metabolic derangements, heart failure, renal failure, respiratory failure, DIC, diabetes, shock, tumor, trauma, sepsis, hepatic disease).

cAG, corrected anion gap; ΔcAG, changes in corrected anion gap; ICU, intensive care unit; OR, odds ratio; CI, confidence interval; APACHE Ⅳ, Acute Physiology and Chronic Health Evaluation Ⅳ; DIC, disseminated intravascular coagulation.

^a^ $\Delta cAG=\frac{\left|final\;cAG\;‐\;initial\;cAG\right|}{initial\;cAG}\times 100\%$ .

^b^ ΔcAG/10 employed to multivariable analysis as a continuous variable.

### 3.3 Sensitivity and subgroup analyses

**FIGURE 3:**
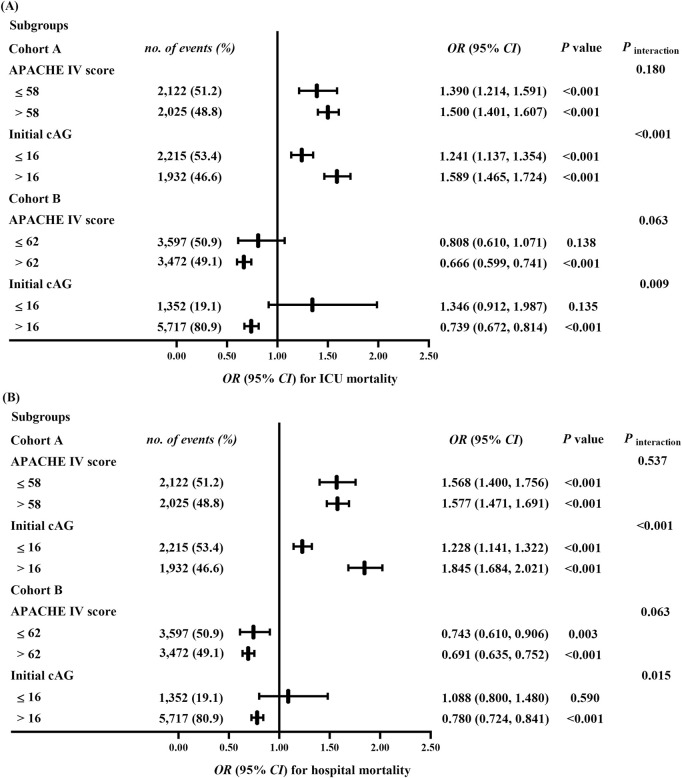
Sensitivity analyses for the association between the ΔcAG^a^ and ICU **(A)** and hospital **(B)** mortalities. All multivariable models were adjusted for demographic information (age [category], sex, ethnicity); APACHE Ⅳ score (except for the APACHE IV score subgroup), biochemical indicators (pH, serum creatinine, lactate, and initial cAG [except for the initial cAG subgroup]); time interval (the hours between the final and the initial cAG measurement); treatments (catecholamine, dialysis, mechanical ventilation, fluid resuscitation, diuretic); clinical comorbidities (metabolic derangements, heart failure, renal failure, respiratory failure, DIC, Diabetes, shock, tumor, trauma, sepsis, hepatic disease). cAG, corrected anion gap; ΔcAG, changes in corrected anion gap, ICU, intensive care unit; OR, odds ratio; CI, confidence interval; APACHE Ⅳ, Acute Physiology and Chronic Health Evaluation Ⅳ; DIC, disseminated intravascular coagulation. ^a^: $\Delta cAG=\frac{\left|final\;cAG\;‐\;initial\;cAG\right|}{initial\;cAG}\times 100\%$ , ΔcAG/10 employed to multivariable analysis as a continuous variable.

We further analyzed the associations between ΔcAG (as a continuous variable) and mortality in predefined subgroups. In cohort A, no significant interaction was observed between the APACHE Ⅳ score (category) and ΔcAG for ICU (*P*_interaction_ = 0.180) or hospital mortality (*P*_interaction_ = 0.537) (Figure 3). In the subgroup with APACHE Ⅳ score ≤58, for every 10% increase in ΔcAG, the risks of ICU and hospital mortalities increased by 39.0% (1.390 [1.214, 1.591]) and 56.8% (1.568 [1.400, 1.756]), respectively. Similarly, in the APACHE Ⅳ score >58 subgroup, the risks increased by 50.0% (1.500 [1.401, 1.607]) and 57.7% (1.577 [1.471, 1.691]), respectively. Furthermore, significant interactions were found between the initial cAG (category) and ΔcAG for ICU and hospital mortalities risks (*P*_interaction_ <0.001, *P*_interaction_ <0.001). A 10% increase in ΔcAG was associated with heightened risks of ICU and hospital mortalities by 24.1% (1.241 [1.137, 1.354]), and 22.8% (1.228 [1.141, 1.322]), respectively, in the initial cAG ≤16 mEq/L subgroup, as well as 58.9% (1.589 [1.465, 1.724]) and 84.5% (1.845 [1.684, 2.021]) in the initial cAG >16 mEq/L subgroup.

In cohort B, we observed no significant interactions between ΔcAG and APACHE IV scores regarding ICU and hospital mortality risks (*P*_interaction_ = 0.063, *P*_interaction_ = 0.063) (Figure 3). In the APACHE Ⅳ score ≤62 subgroup, there was no statistically significant association between ΔcAG and ICU mortality (*P* = 0.138). However, the risk of hospital mortality decreased by 25.7% (0.743 [0.610, 0.906]) for every 10% increase in ΔcAG. In the APACHE Ⅳ score >62 subgroup, a reduction was observed in the risks of ICU and hospital mortalities by 33.4% (0.666 [0.599, 0.741]) and 30.1% (0.691 [0.635, 0.752]), respectively. Moreover, significant interactions were found between the initial cAG and ΔcAG for risks of mortalities (*P*_interaction_ = 0.009, *P*_interaction_ = 0.015). No statistically significant association was found between ΔcAG and ICU or hospital mortality (*P* = 0.135, *P* = 0.590) in the initial cAG ≤16 mEq/L subgroup. Conversely, in the initial cAG >16 mEq/L subgroup, a 10% increase in ΔcAG was associated with decreased risks of ICU and hospital mortalities by 26.1% (0.739 [0.672, 0.814]) and 22.0% (0.780 [0.724, 0.841]), respectively.

No significant interaction was observed between ΔcAG and the time interval (between the initial and final cAG) for ICU and hospital mortalities risks in either cohort A (*P*_interaction_ = 0.490 and *P*_interaction_ = 0.118, respectively) or cohort B (*P*_interaction_ = 0.054 and *P*_interaction_ = 0.249, respectively). The relationship between ΔcAG and mortality remained consistent in subgroup analyses (Supplementary Table 3). After applying Bonferroni correction for multiple comparisons, the associations between ΔcAG and mortality remained robust across all predefined subgroups.

## 4 Discussion

In this study, we performed a large-scale multicenter retrospective cohort study among critically ill patients to explore the association between mortality and changes in cAG during ICU hospitalization. For patients with an elevated final cAG, we observed that each 10% increase in ΔcAG was associated with 46.1% and 55.5% increased risks of ICU and hospital mortalities, respectively. Notably, this association remained robust across patients with different APACHE IV scores, time interval and initial cAG levels. Remarkably, a reduction in final cAG compared to the initial value exhibited a beneficial effect on ICU and hospital mortalities, particularly among patients with severe illness (APACHE IV score >62) and higher initial cAG level (>16 mEq/L).

The AG is estimated by assessing the differences between serum cations (Na^+^ and K^+^) and anions (Cl^−^ and HCO_3_^−^) (Seifter, 2014). Typically, albumin and phosphate are the primary contributors to this “gap”, with minor contributions from sulfate and lactate, usually less than 2 mEq/L (Al-Jaghbeer and Kellum, 2015). In this study, we excluded patients whose albumin values were not recorded within 24 h of the AG to ensure the accuracy of the cAG value. Previous studies have demonstrated a correlation between elevated AG (Chen et al., 2022; Cheng et al., 2020; Gao et al., 2021; Zhu et al., 2023)/cAG (Giri, et al., 2025; Hu et al., 2023; Hu, et al., 2021; Lee, et al., 2019; Zhao, et al., 2023) values at ICU admission and increased mortality across various clinical scenarios. However, a retrospective study of 1,470 critically ill surgical patients found that AG values at admission were not predictive of mortality (0.940 [0.009, 95.900]) (Martin et al., 2013). Similarly, another retrospective study involving 300 critically ill adult patients admitted to the ICU reported modest AUROC curves for cAG and AG in predicting mortality: 0.67 (0.60, 0.74) and 0.66 (0.59, 0.73), respectively (Rocktaeschel, et al., 2003). Furthermore, several studies have found no significant difference in AG (Attanà et al., 2013; Boniatti et al., 2011) or cAG (Boniatti, et al., 2011) between survivors and non-survivors at ICU admission. Notably, in critically ill COVID-19 patients, AG values showed no intergroup difference (survivors vs. non-survivors), yet non-survivors exhibited a significant AG variability (Pulgar-Sánchez et al., 2024). Our results showed an inconsistency between the initial and final cAG levels. Interventions such as renal replacement therapy (RRT) (Cerdá, et al., 2012) and mechanical ventilation (Carrillo Alvarez, 2003) can alter cAG levels during ICU hospitalization. Additionally, critical illnesses like septic shock can induce tissue hypoxia and elevate lactate levels (Kraut and Madias, 2014), thereby increasing cAG values. Therefore, relying on a single cAG measurement fails to reflect the dynamic clinical status of ICU patients. Considering changes in cAG over time could enhance its predictive capability for patient prognosis.

Several studies have focused on the correlation between dynamic AG value and critically ill patient outcomes. A meta-analysis of nine studies involving 12,497 patients indicated that dynamic AG could strongly predict mortality by considering the extent and trends of acid-base disturbance (Glasmacher and Stones, 2016). Lipnick, et al. (2013) revealed a relationship between an increased ΔAG, calculated as the difference between ICU admission AG and prehospital value, and all-cause mortality. Xie, et al. (2022) investigated the association between hospital mortality and ΔAG (ΔAG = AG_max_ - AG_min_) within the initial 3 days post-admission to cardiothoracic surgery recovery unit, revealing ΔAG as a potential prognostic indicator for hospital mortality and 90-day survival. These studies highlight the predictive ability of ΔAG for mortality in critically ill patients, with primary focus on short-term ICU admission. This study introduces ΔcAG, which is defined as the percentage difference between the final cAG and initial value measured at ICU admission, encompassing the entire duration of ICU stay. It provides a more interpretable measure of metabolic progression across patients with varying initial cAG levels. The median time intervals for ΔcAG were 97 and 101 h in cohorts A and B, respectively. In cohort A, each 10% increase in ΔcAG was associated with increasing ICU and hospital mortalities by 46.1% and 55.5%, respectively. Furthermore, among patients in cohort B, the ICU and hospital mortality decreased by 31.4% and 29.4%, respectively, for each 10% increase in ΔcAG. These findings underscore the importance of monitoring cAG trends level during the ICU stay, as an elevation in cAG may signal an increased risk of mortality risk, warranting heightened clinical vigilance.

The APACHE Ⅳ score is a crucial tool for assessing disease severity in ICU patients, with higher scores indicating poorer clinical condition (Ko et al., 2018; Zimmerman et al., 2006). The accumulation of acid could significantly affect cellular function and increase morbidity and mortality (Gunnerson et al., 2006; Kraut and Kurtz, 2005). To account for the influence of disease severity and initial cAG levels, this study divided patients into two subgroups for further investigation. Among different subgroups based on APACHE Ⅳ scores (≤58 vs. >58) or initial cAG levels (≤16 mEq/L vs. >16 mEq/L), ΔcAG consistently demonstrated a strong association with an increased mortality risk in cohort A. In addition, in cohort B, patients with higher APACHE Ⅳ scores (>62) and elevated initial cAG levels (>16 mEq/L) exhibited a significant survival advantage in the ICU. These findings suggest that, regardless of the severity of the illness, elevating cAG is hazardous, while a reduction of cAG might prove benefits, particularly for ICU patients with a more serious illness.

The mechanisms underlying the correlation between ΔcAG and mortality in critically ill patients are not yet well understood. Firstly, an elevated cAG signifies the buffering of H^+^ derived from nonvolatile acids by HCO_3_^−^, leading to HCO_3_^−^ depletion and H^+^ accumulation. This adversely affects cardiac Ca^2+^ signaling and myofilament sensitivity, thereby reducing contractility (Orchard and Kentish, 1990). Intracellular H^+^ buffering promotes K^+^ efflux, increasing arrhythmia risk (Orchard and Cingolani, 1994). In shock, H^+^ weakens catecholamine response, and lactic acidosis induces vascular smooth muscle relaxation *via* the opening of ATP-sensitive potassium channels (Kimmoun et al., 2015). H^+^ also enhances γ-aminobutyric acid neurotransmission, leading to consciousness depression and blunted respiratory drive, and triggers the release of proinflammatory cytokine, which amplifies systemic inflammation (Coppola et al., 2021). Attenuation of elevated cAG may offer survival benefits in critically ill patients. Clinicians have employed targeted interventions to reduce cAG levels. For instance, they used RRT to normalize AG imbalance in the ICU(Cerdá, et al., 2012; Yagi and Fujii, 2021), administrated of sodium bicarbonate to reduce cAG value (Fujii et al., 2019) in patients with cardiopulmonary resuscitation (Bar-Joseph et al., 2005), gastrointestinal disorders (Jung et al., 2019), and acute kidney injure (Jaber et al., 2018; Zhang et al., 2018), and carried out fluid resuscitation to promote lactate clearance, thereby enhancing the outcomes in sepsis shock (Evans et al., 2021). Secondly, a potential reverse causality between an elevated final cAG and progression of critical illness, such as acute kidney injury, diabetic ketoacidosis, and lactic acidosis (Kraut and Madias, 2010). These pathophysiological processes may establish a feedback loop, where metabolic disturbances could both contribute to and result from disease progression. The interplay may precipitate organ failure and life-threatening circumstances.

This study has several limitations that should be acknowledged. Firstly, as a retrospective observational cohort study based on real-world data, inherent bias such as selection bias, measurement bias, and confounding are inevitable (Hong, 2021). Although we attempted to mitigates these biased by incorporating know confounding factors into the logistic regression model, residual or unmeasured confounders might still influence the observed association between ΔcAG and mortality. Secondly, although this study revealed a significant association between ΔcAG and mortality in critically ill patients, the retrospective nature restricts our ability to establish causality or infer definitive clinical thresholds. Thirdly, the reliance on albumin measurement introduces a potential selection bias, as patients included in our study may represent a subgroup receiving more intensive metabolic monitoring. This could limit the generalizability of our findings to broader critically ill populations. Therefore, given these limitations, future extensive multicenter prospective studies should be conducted to further validate the role of ΔcAG as a prognostic factor for predicting clinical outcomes.

## 5 Conclusion

In this study, we provide evidence that an increase in the cAG relative to the initial measurement after ICU admission is strongly associated with an elevated mortality risk in critically ill patients. These findings suggest the importance of monitoring dynamic changes in cAG over time, rather than relying solely on the initial cAG value. Nonetheless, further research, especially rigorously designed prospective studies, is needed to evaluate and verify these findings.

## Data Availability

Publicly available datasets were analyzed in this study. This data can be found here: https://physionet.org/content/eicu-crd/.

## Abbreviations

AG, anion gap; cAG, corrected AG; ICU, intensive care unit; AUROC, areas under the receiver operating characteristic curve; ΔcAG, change in cAG; cAG_Diff_, absolute change in cAG; eICU-CRD, eICU Collaborative Research Database; APACHE Ⅳ, Acute Physiology and Chronic Health Evaluation Ⅳ; ICD-9, International Classification of Diseases-9; DIC, disseminated intravascular coagulation; Na^+^, sodium; K^+^, potassium; Cl^-^, chloride; HCO_3_^−^, bicarbonate; IQRs, interquartile ranges; MICE, multivariate imputation by chained equation; RCS, restricted cubic spline; OR, odds ratio; CI, confidence interval; RRT, renal replacement therapy.

## References

[B1] Achanti A. Szerlip H. M. (2023). Acid-base disorders in the critically ill patient. Clin. J. Am. Soc. Nephrol. 18 (1), 102–112. 10.2215/cjn.04500422 35998977 PMC10101555

[B2] Al-Jaghbeer M. Kellum J. A. (2015). Acid-base disturbances in intensive care patients: etiology, pathophysiology and treatment. Nephrol. Dial. Transpl. 30 (7), 1104–1111. 10.1093/ndt/gfu289 25213433

[B3] Attanà P. Lazzeri C. Chiostri M. Picariello C. Gensini G. F. Valente S. (2013). Strong-ion gap approach in patients with cardiogenic shock following ST-elevation myocardial infarction. Acute Card. Care 15 (3), 58–62. 10.3109/17482941.2013.776691 23806089

[B4] Bar-Joseph G. Abramson N. S. Kelsey S. F. Mashiach T. Craig M. T. Safar P. (2005). Improved resuscitation outcome in emergency medical systems with increased usage of sodium bicarbonate during cardiopulmonary resuscitation. Acta Anaesthesiol. Scand. 49 (1), 6–15. 10.1111/j.1399-6576.2005.00572.x 15675975

[B5] Boniatti M. M. Cardoso P. R. Castilho R. K. Vieira S. R. (2011). Is hyperchloremia associated with mortality in critically ill patients? A prospective cohort study. J. Crit. Care 26 (2), 175–179. 10.1016/j.jcrc.2010.04.013 20619601

[B6] Carrillo Alvarez A. (2003). Mechanical ventilation monitoring: gas analysis and acid base balance. An Pediatr (Barc). 59 (3), 252–259. 10.1016/s1695-4033(03)78175-2 12975118

[B7] Cerdá J. Tolwani A. J. Warnock D. G. (2012). Critical care nephrology: management of acid-base disorders with CRRT. Kidney Int. 82 (1), 9–18. 10.1038/ki.2011.243 21814173

[B8] Chen J. Dai C. Yang Y. Wang Y. Zeng R. Li B. (2022). The association between anion gap and in-hospital mortality of post-cardiac arrest patients: a retrospective study. Sci. Rep. 12 (1), 7405. 10.1038/s41598-022-11081-3 35524151 PMC9076652

[B9] Cheng B. Li D. Gong Y. Ying B. Wang B. (2020). Serum anion gap predicts all-cause mortality in critically ill patients with acute kidney injury: analysis of the MIMIC-III database. Dis. Markers 2020, 6501272. 10.1155/2020/6501272 32051697 PMC6995483

[B10] Coppola S. Caccioppola A. Froio S. Chiumello D. (2021). Sodium bicarbonate in different critically ill conditions: from Physiology to clinical practice. Anesthesiology 134 (5), 774–783. 10.1097/aln.0000000000003733 33721887

[B11] Evans L. Rhodes A. Alhazzani W. Antonelli M. Coopersmith C. M. French C. (2021). Surviving sepsis campaign: international guidelines for management of sepsis and septic shock 2021. Intensive Care Med. 47 (11), 1181–1247. 10.1007/s00134-021-06506-y 34599691 PMC8486643

[B12] Figge J. Jabor A. Kazda A. Fencl V. (1998). Anion gap and hypoalbuminemia. Crit. Care Med. 26 (11), 1807–1810. 10.1097/00003246-199811000-00019 9824071

[B13] Fujii T. Udy A. Licari E. Romero L. Bellomo R. (2019). Sodium bicarbonate therapy for critically ill patients with metabolic acidosis: a scoping and a systematic review. J. Crit. Care 51, 184–191. 10.1016/j.jcrc.2019.02.027 30852347

[B14] Gao Y. Hong Z. Shen R. Zhang S. You G. Chen J. (2021). Association between anion gap and mortality of aortic aneurysm in intensive care unit after open surgery. BMC Cardiovasc. Disord. 21 (1), 458. 10.1186/s12872-021-02263-4 34556051 PMC8459533

[B15] Giri M. Puri A. Huang L. Guo S. (2025). Association between albumin corrected anion gap and in-hospital mortality in critically ill patients with chronic obstructive pulmonary disease. Ther. Adv. Respir. Dis. 19, 17534666251315352. 10.1177/17534666251315352 39866132 PMC11770725

[B16] Glasmacher S. A. Stones W. (2016). Anion gap as a prognostic tool for risk stratification in critically ill patients - a systematic review and meta-analysis. BMC Anesthesiol. 16 (1), 68. 10.1186/s12871-016-0241-y 27577038 PMC5006450

[B17] Goldstein M. B. Halperin M. L. (2010). Fluid, electrolyte and acid-base Physiology, 4th edition - a problem-based approach.

[B18] Goodkin D. A. Krishna G. G. Narins R. G. (1984). The role of the anion gap in detecting and managing mixed metabolic acid-base disorders. Clin. Endocrinol. Metab. 13 (2), 333–349. 10.1016/s0300-595x(84)80025-0 6488577

[B19] Gunnerson K. J. Saul M. He S. Kellum J. A. (2006). Lactate versus non-lactate metabolic acidosis: a retrospective outcome evaluation of critically ill patients. Crit. Care 10 (1), R22. 10.1186/cc3987 16507145 PMC1550830

[B20] Hatherill M. Waggie Z. Purves L. Reynolds L. Argent A. (2002). Correction of the anion gap for albumin in order to detect occult tissue anions in shock. Arch. Dis. Child. 87 (6), 526–529. 10.1136/adc.87.6.526 12456555 PMC1755806

[B21] Hong J. C. (2021). Strategies to turn real-world data into real-world knowledge. JAMA Netw. Open 4 (10), e2128045. 10.1001/jamanetworkopen.2021.28045 34618043

[B23] Hu B. Zhong L. Yuan M. Min J. Ye L. Lu J. (2023). Elevated albumin corrected anion gap is associated with poor in-hospital prognosis in patients with cardiac arrest: a retrospective study based on MIMIC-IV database. Front. Cardiovasc. Med. 10, 1099003. 10.3389/fcvm.2023.1099003 37034339 PMC10076801

[B24] Hu T. Zhang Z. Jiang Y. (2021). Albumin corrected anion gap for predicting in-hospital mortality among intensive care patients with sepsis: a retrospective propensity score matching analysis. Clin. Chim. Acta 521, 272–277. 10.1016/j.cca.2021.07.021 34303712

[B25] Jaber S. Paugam C. Futier E. Lefrant J. Y. Lasocki S. Lescot T. (2018). Sodium bicarbonate therapy for patients with severe metabolic acidaemia in the intensive care unit (BICAR-ICU): a multicentre, open-label, randomised controlled, phase 3 trial. Lancet 392 (10141), 31–40. 10.1016/s0140-6736(18)31080-8 29910040

[B26] Jung B. Martinez M. Claessens Y. E. Darmon M. Klouche K. Lautrette A. (2019). Diagnosis and management of metabolic acidosis: guidelines from a French expert panel. Ann. Intensive Care 9 (1), 92. 10.1186/s13613-019-0563-2 31418093 PMC6695455

[B28] Kimmoun A. Novy E. Auchet T. Ducrocq N. Levy B. (2015). Hemodynamic consequences of severe lactic acidosis in shock states: from bench to bedside. Crit. Care 19 (1), 175. 10.1186/s13054-015-0896-7 25887061 PMC4391479

[B29] Ko M. Shim M. Lee S. M. Kim Y. Yoon S. (2018). Performance of Apache IV in medical intensive care unit patients: comparisons with Apache II, saps 3, and MPM(0) III. Acute Crit. Care 33 (4), 216–221. 10.4266/acc.2018.00178 31723888 PMC6849024

[B30] Kraut J. A. Kurtz I. (2005). Metabolic acidosis of CKD: diagnosis, clinical characteristics, and treatment. Am. J. Kidney Dis. 45 (6), 978–993. 10.1053/j.ajkd.2005.03.003 15957126

[B31] Kraut J. A. Madias N. E. (2010). Metabolic acidosis: pathophysiology, diagnosis and management. Nat. Rev. Nephrol. 6 (5), 274–285. 10.1038/nrneph.2010.33 20308999

[B32] Kraut J. A. Madias N. E. (2014). Lactic acidosis. N. Engl. J. Med. 371 (24), 2309–2319. 10.1056/NEJMra1309483 25494270

[B33] Kutner M. H. Nachtsheim C. J. Neter J. Wasserman W. (2004). Applied linear regression model. New York: McGraw-Hill/Irwin. 10.2307/1269508

[B34] Lee S. B. Kim D. H. Kim T. Lee S. H. Jeong J. H. Kim S. C. (2019). Anion gap and base deficit are predictors of mortality in acute pesticide poisoning. Hum. Exp. Toxicol. 38 (2), 185–192. 10.1177/0960327118788146 30001645

[B35] Lipnick M. S. Braun A. B. Cheung J. T. Gibbons F. K. Christopher K. B. (2013). The difference between critical care initiation anion gap and prehospital admission anion gap is predictive of mortality in critical illness. Crit. Care Med. 41 (1), 49–59. 10.1097/CCM.0b013e31826764cd 23190721

[B36] Martin J. Blobner M. Busch R. Moser N. Kochs E. Luppa P. B. (2013). Point-of-care testing on admission to the intensive care unit: lactate and glucose independently predict mortality. Clin. Chem. Lab. Med. 51 (2), 405–412. 10.1515/cclm-2012-0258 22987833

[B39] Oh M. S. (2011). Evaluation of renal function, water, electrolytes, and acid-base balance. Henry's. Clin. Diagnosis Manag. by Laboratory Methods, 169–192. 10.1016/b978-1-4377-0974-2.00014-2

[B40] Orchard C. H. Cingolani H. E. (1994). Acidosis and arrhythmias in cardiac muscle. Cardiovasc. Res. 28 (9), 1312–1319. 10.1093/cvr/28.9.1312 7954638

[B41] Orchard C. H. Kentish J. C. (1990). Effects of changes of pH on the contractile function of cardiac muscle. Am. J. Physiol. 258 (6 Pt 1), C967–C981. 10.1152/ajpcell.1990.258.6.C967 2193525

[B42] Pollard T. J. Johnson A. E. W. Raffa J. D. Celi L. A. Mark R. G. Badawi O. (2018). The eICU Collaborative Research Database, a freely available multi-center database for critical care research. Sci. Data 5, 180178. 10.1038/sdata.2018.178 30204154 PMC6132188

[B43] Pulgar-Sánchez M. Chamorro K. Casella C. Ballaz S. J. (2024). Insights into the baseline blood pH homeostasis at admission and the risk of in-hospital mortality in COVID-19 patients. Biomark. Med. 18 (19), 795–800. 10.1080/17520363.2024.2395800 39255012 PMC11497984

[B44] Rocktaeschel J. Morimatsu H. Uchino S. Bellomo R. (2003). Unmeasured anions in critically ill patients: can they predict mortality? Crit. Care Med. 31 (8), 2131–2136. 10.1097/01.ccm.0000079819.27515.8e 12973170

[B45] Seifter J. L. (2014). Integration of acid-base and electrolyte disorders. N. Engl. J. Med. 371 (19), 1821–1831. 10.1056/NEJMra1215672 25372090

[B46] Xie K. Zheng C. Wang G. M. Diao Y. F. Luo C. Wang E. (2022). Association between delta anion gap and hospital mortality for patients in cardiothoracic surgery recovery unit: a retrospective cohort study. BMC Surg. 22 (1), 186. 10.1186/s12893-022-01625-9 35568886 PMC9107697

[B47] Yagi K. Fujii T. (2021). Management of acute metabolic acidosis in the ICU: sodium bicarbonate and renal replacement therapy. Crit. Care 25 (1), 314. 10.1186/s13054-021-03677-4 34461963 PMC8406840

[B48] Zhang Z. (2016). Multiple imputation with multivariate imputation by chained equation (MICE) package. Ann. Transl. Med. 4 (2), 30. 10.3978/j.issn.2305-5839.2015.12.63 26889483 PMC4731595

[B49] Zhang Z. Zhu C. Mo L. Hong Y. (2018). Effectiveness of sodium bicarbonate infusion on mortality in septic patients with metabolic acidosis. Intensive Care Med. 44 (11), 1888–1895. 10.1007/s00134-018-5379-2 30255318

[B50] Zhao B. Li Y. Lang X. Fang S. Li Z. Li L. (2023). Increased serum albumin corrected anion gap levels are associated with increased incidence of new-onset HF and poor prognosis in patients with acute myocardial infarction. Clin. Chim. Acta. 544, 117354. 10.1016/j.cca.2023.117354 37076098

[B51] Zhu Y. He Z. Jin Y. Zhu S. Xu W. Li B. (2023). Serum anion gap level predicts all-cause mortality in septic patients: a retrospective study based on the mimic III database. J. Intensive Care Med. 38 (4), 349–357. 10.1177/08850666221123483 36066040

[B52] Zimmerman J. E. Kramer A. A. McNair D. S. Malila F. M. (2006). Acute Physiology and Chronic Health Evaluation (Apache) IV: hospital mortality assessment for today's critically ill patients. Crit. Care Med. 34 (5), 1297–1310. 10.1097/01.ccm.0000215112.84523.f0 16540951

