# Supplementary material for: Association between changes in corrected anion gap and mortality among critically ill patients during ICU stay: a multicenter observational study
Source: Front Physiol. 2025 Jun 25;16:1469985. doi: 10.3389/fphys.2025.1469985 (PMC12238759; doi:10.3389/fphys.2025.1469985)
Supplement: Supplementary file 1 [file Supplementaryfile1.doc]

**Table 1. Baseline characteristics of Cohort B.**

| **Characteristics** | **Entire population**  **(N = 7,069)** | **ΔcAG (%)** | | | |  |
| --- | --- | --- | --- | --- | --- | --- |
|  |  | **Q1: ≤ 7.48%**  **(N = 1,768)** | **Q2: 7.48–15.05%**  **(N = 1,767)** | **Q4: 15.05–24.70%**  **(N = 1,768)** | **Q4: > 24.70%**  **(N = 1,766)** | ***P* value** |
| **Age n (%)** |  |  |  |  |  | <0.001 |
| **≤65 y** | 3,877 (54.8) | 919 (52.0) | 914 (51.7) | 971 (54.9) | 1,073 (60.8) |  |
| **>65 y** | 3,192 (45.2) | 849 (48.0) | 853 (48.3) | 797 (45.1) | 693 (39.2) |  |
| **Sex n (%)** |  |  |  |  |  | 0.591 |
| **Male** | 3,876 (54.8) | 992 (56.1) | 956 (54.1) | 957 (54.1) | 971 (55.0) |  |
| **Female** | 3,193 (45.2) | 776 (43.9) | 811 (45.9) | 811 (45.9) | 795 (45.00) |  |
| **Ethnicity n (%)** |  |  |  |  |  | 0.947 |
| **Caucasian** | 5,452 (77.1) | 1,362 (77.0) | 1,367 (77.4) | 1,369 (77.4) | 1,354 (76.7) |  |
| **Others/Unknown** | 1,617 (22.9) | 406 (23.0) | 400 (22.6) | 399 (22.6) | 412 (23.3) |  |
| **APACHE Ⅳ score** | 62 (46–81) | 58 (42–76) | 58 (44–76) | 63 (46–83) | 69 (53–89) | <0.001 |
| **Initial cAG (mEq/L)** | 19.10 (16.65–22.15) | 17.55 (15.45–19.60) | 18.15 (16.10–20.50) | 19.45 (17.10–22.05) | 22.35 (19.40–26.60) | <0.001 |
| **Time Interval (hours)** | 101 (55–194) | 87 (49–167) | 96 (50–167) | 108 (59–198) | 132 (72–241) |  |
| **PH value** | 7.37 (7.30–7.42) | 7.38 (7.32–7.42) | 7.38 (7.32–7.43) | 7.37 (7.30–7.42) | 7.34 (7.25–7.40) | <0.001 |
| **Lactate (mmol/L)** | 1.70 (1.10–2.80) | 1.50 (1.00–2.30) | 1.60 (1.00–2.50) | 1.70 (1.10–2.70) | 2.00 (1.20–4.00) | <0.001 |
| **Creatinine (mg/dL)** | 1.18 (0.80–2.10) | 1.07 (0.76–1.80) | 1.08 (0.75–1.74) | 1.15 (0.79–2.12) | 1.50 (0.91–2.80) | <0.001 |
| **Treatments n (%)** |  |  |  |  |  |  |
| **Catecholamine** | 1,933 (27.3) | 394 (22.3) | 433 (24.5) | 487 (27.5) | 619 (35.1) | <0.001 |
| **Dialysis** | 558 (7.9) | 113 (6.4) | 109 (6.2) | 126 (7.1) | 210 (11.9) | <0.001 |
| **Mechanical Ventilation** | 3267 (46.2) | 744 (42.1) | 803 (45.4) | 833 (47.1) | 887 (50.2) | <0.001 |
| **Fluid Resuscitation** | 509 (7.2) | 116 (6.6) | 103 (5.8) | 112 (6.3) | 178 (10.1) | <0.001 |
| **Diuretic** | 1593 (22.5) | 360 (20.4) | 361 (20.4) | 451 (25.5) | 421 (23.8) | <0.001 |
| **Diagnoses n (%)** |  |  |  |  |  |  |
| **Metabolic derangements** | 577 (8.2) | 80 (4.5) | 87 (4.9) | 127 (7.2) | 283 (16.0) | <0.001 |
| **Heart Failure** | 509 (7.2) | 125 (7.1) | 117 (6.6) | 142 (8.0) | 125 (7.1) | 0.424 |
| **Renal Failure** | 1,254 (17.7) | 230 (13.0) | 237 (13.4) | 312 (17.6) | 475 (26.9) | <0.001 |
| **Respiratory Failure** | 1,714 (24.2) | 389 (22.0) | 401 (22.7) | 445 (25.2) | 479 (27.1) | 0.001 |
| **DIC** | 23 (0.30) | 4 (0.2) | 4 (0.2) | 7 (0.4) | 8 (0.5) | 0.559 |
| **Diabetes** | 898 (12.7) | 189 (10.7) | 195 (11.0) | 191 (10.8) | 323 (18.3) | <0.001 |
| **Shock** | 1,944 (27.5) | 419 (23.7) | 439 (24.8) | 508 (28.7) | 578 (32.7) | <0.001 |
| **Tumor** | 381 (5.4) | 117 (6.6) | 100 (5.7) | 91 (5.1) | 73 (4.1) | 0.011 |
| **Trauma** | 395 (5.6) | 113 (6.4) | 100 (5.7) | 98 (5.5) | 84 (4.8) | 0.212 |
| **Sepsis** | 1,645 (23.3) | 362 (20.5) | 382 (21.6) | 440 (24.9) | 461 (26.1) | <0.001 |
| **Hepatic Disease** | 629 (8.9) | 142 (8.0) | 148 (8.4) | 158 (8.9) | 181 (10.2) | 0.102 |
| **ICU Mortality** | 508 (7.2) | 139 (7.9) | 108 (6.1) | 123 (7.0) | 138 (7.8) | 0.142 |
| **Hospital Mortality** | 864 (12.2) | 223 (12.6) | 211 (11.9) | 208 (11.8) | 222 (12.6) | 0.819 |

cAG, corrected anion gap; ΔcAG, change in corrected anion gap, APACHE Ⅳ, Acute Physiology and Chronic Health Evaluation Ⅳ; DIC, disseminated intravascular coagulation; ICU, intensive care unit. Time Interval, the time interval between the final and the initial cAG measurement; *AG* = ([*Na*^+^] mEq/L + [*K*^+^] mEq/L) - ([*Cl*^-^] mEq/L + [*HCO_3_*^-^] mEq/L); *cAG* = *AG* + 2.5 × [4.4 - *albumin* (g/dL)];
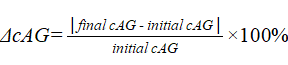
.

**Table 2. Multivariable analysis of the association between cAG_Diff_ and mortality.**

| **cAG_Diff_** ^a^ **Variable** | **ICU Mortality** | |  | **Hospital Mortality** | |
| --- | --- | --- | --- | --- | --- |
|  | ***OR* (95% *CI*)** | ***P* Value** |  | ***OR* (95% *CI*)** | ***P* Value** |
| **Cohort A** |  |  |  |  |  |
| Continuous variable | 1.265 (1.222, 1.310) | < 0.001 |  | 1.364 (1.312, 1.418) | < 0.001 |
| Categorical variable |  |  |  |  |  |
| Q1 | 1 (Ref) |  |  | 1 (Ref) |  |
| Q2 | 1.347 (0.923, 1.966) | 0.123 |  | 1.383 (1.007, 1.899) | 0.045 |
| Q3 | 1.878 (1.293, 2.727) | 0.001 |  | 2.210 (1.618, 3.020) | < 0.001 |
| Q4 | 5.821 (4.164, 8.138) | < 0.001 |  | 7.048 (5.259, 9.444) | < 0.001 |
| **Cohort B** |  |  |  |  |  |
| Continuous variable | 0.821 (0.789, 0.855) | < 0.001 |  | 0.836 (0.809, 0.864) | < 0.001 |
| Categorical variable |  |  |  |  |  |
| Q1 | 1 (Ref) |  |  | 1 (Ref) |  |
| Q2 | 0.600 (0.442, 0.813) | 0.001 |  | 0.783 (0.619, 0.989) | 0.040 |
| Q3 | 0.567 (0.421, 0.764) | < 0.001 |  | 0.624 (0.492, 0.791) | 0.040 |
| Q4 | 0.370 (0.264, 0.519) | < 0.001 |  | 0.422 (0.322, 0.553) | 0.040 |

**Multivariable model:** adjusted for demographic information (age [category], sex, ethnicity); APACHE Ⅳ score, biochemical indicators (pH, serum creatinine, lactate, and initial cAG); time interval (the hours between the final and the initial cAG measurement); treatments (catecholamine, dialysis, mechanical ventilation, fluid resuscitation, diuretic); clinical comorbidities (metabolic derangements, heart failure, renal failure, respiratory failure, DIC, Diabetes, shock, tumor, trauma, sepsis, hepatic disease).

cAG, corrected anion gap; ΔcAG, changes in corrected anion gap, ICU, intensive care unit; OR, odds ratio; CI, confidence interval; APACHE Ⅳ, Acute Physiology and Chronic Health Evaluation Ⅳ; DIC, disseminated intravascular coagulation.

^a^: *cAG_Diff_* = ∣*final cAG – initial cAG*∣

**Table 3. Subgroup analyses for the association between ΔcAG and mortality stratified by time interval between the initial and final records*.***

| **ΔcAG** ^a^ **Variable** | **ICU Mortality** | | **Hospital Mortality** | |
| --- | --- | --- | --- | --- |
|  | ***OR* (95% *CI*)** | ***P* Value** | ***OR* (95% *CI*)** | ***P* Value** |
| **Cohort A** |  |  |  |  |
| ≤ 97 hours | 1.453 (1.320, 1.601) | < 0.001 | 1.644 (1.482, 1.824) | < 0.001 |
| > 97 hours | 1.435 (1.331, 1.548) | < 0.001 | 1.494 (1.391, 1.605) | < 0.001 |
| **Cohort B** |  |  |  |  |
| ≤ 101 hours | 0.674 (0.577, 0.788) | < 0.001 | 0.664 (0.583, 0.757) | < 0.001 |
| > 101 hours | 0.738 (0.642, 0.848) | <0.001 | 0.774 (0.698, 0.859) | < 0.001 |

**Multivariable model:** adjusted for demographic information (age [category], sex, ethnicity); APACHE Ⅳ score, biochemical indicators (pH, serum creatinine, lactate, and initial cAG); treatments (catecholamine, dialysis, mechanical ventilation, fluid resuscitation, diuretic); clinical comorbidities (metabolic derangements, heart failure, renal failure, respiratory failure, DIC, Diabetes, shock, tumor, trauma, sepsis, hepatic disease).

cAG, corrected anion gap; ΔcAG, changes in corrected anion gap, ICU, intensive care unit; OR, odds ratio; CI, confidence interval; APACHE Ⅳ, Acute Physiology and Chronic Health Evaluation Ⅳ; DIC, disseminated intravascular coagulation.

^a^:
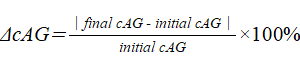
, ΔcAG/10 employed to multivariable analysis as a continuous variable.

**Figure 1 Connection between the initial cAG and final cAG.**

**
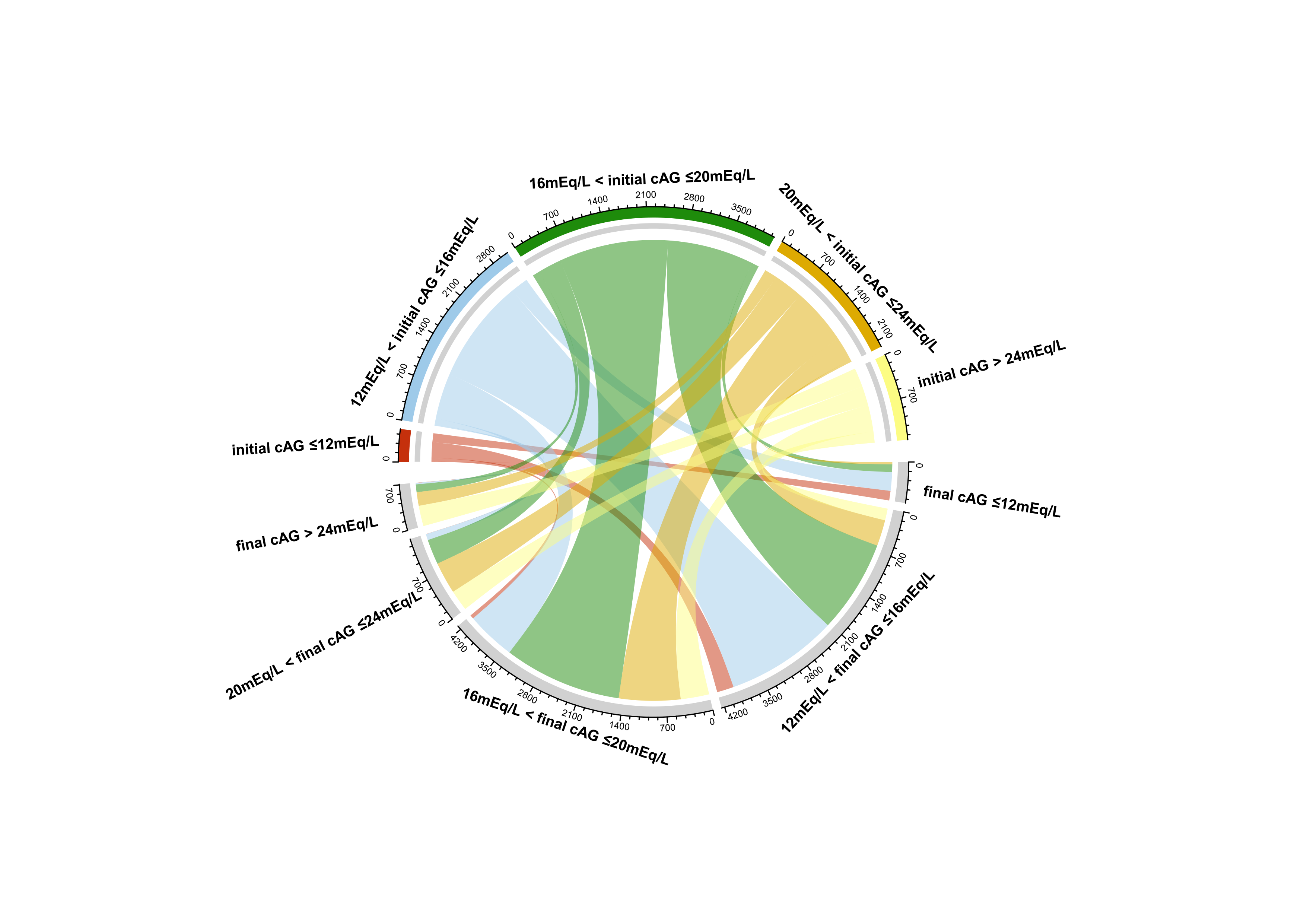
**

A chord diagram shows the difference between the initial cAG and the final cAG for each patient.

cAG corrected anion gap
